# The Strong Activation of p53 Tumor Suppressor Drives the Synthesis of the Enigmatic Isoform of DUSP13 Protein

**DOI:** 10.3390/biomedicines12071449

**Published:** 2024-06-28

**Authors:** Małgorzata Krześniak, Barbara Łasut-Szyszka, Agnieszka Będzińska, Agnieszka Gdowicz-Kłosok, Marek Rusin

**Affiliations:** Center for Translational Research and Molecular Biology of Cancer, Maria Skłodowska-Curie National Research Institute of Oncology, Gliwice Branch, 44-101 Gliwice, Poland; malgorzata.krzesniak@gliwice.nio.gov.pl (M.K.); barbara.lasut-szyszka@gliwice.nio.gov.pl (B.Ł.-S.); agnieszka.bedzinska@gliwice.nio.gov.pl (A.B.); agnieszka.gdowicz-klosok@gliwice.nio.gov.pl (A.G.-K.)

**Keywords:** p53, MDM2, DUSP13, alternative promoter, dual-specificity phosphatase

## Abstract

The p53 tumor suppressor protein activates various sets of genes depending on its covalent modifications, which are controlled by the nature and intensity of cellular stress. We observed that actinomycin D and nutlin-3a (A + N) collaborate in inducing activating phosphorylation of p53. Our recent transcriptomic data demonstrated that these substances strongly synergize in the upregulation of *DUSP13*, a gene with an unusual pattern of expression, coding for obscure phosphatase having two isoforms, one expressed in the testes and the other in skeletal muscles. In cancer cells exposed to A + N, *DUSP13* is expressed from an alternative promoter in the intron, resulting in the expression of an isoform named TMDP-L1. Luciferase reporter tests demonstrated that this promoter is activated by both endogenous and ectopically expressed p53. We demonstrated for the first time that mRNA expressed from this promoter actually produces the protein, which can be detected with Western blotting, in all examined cancer cell lines with wild-type p53 exposed to A + N. In some cell lines, it is also induced by clinically relevant camptothecin, by nutlin-3a acting alone, or by a combination of actinomycin D and other antagonists of p53-MDM2 interaction—idasanutlin or RG7112. This isoform, fused with green fluorescent protein, localizes in the perinuclear region of cells.

## 1. Introduction

The p53 tumor suppressor protein impacts the functioning of a cell by behaving as an activator of at least hundreds of genes. The early-identified p53-regulated genes coded for proteins promoting apoptosis, cell cycle arrest, and DNA repair. However, as many more genes directly activated by p53 have been discovered, it was realized that p53 regulates a wide spectrum of biological functions, including metabolism, aging, angiogenesis, immunity, and more [1,2,3]. The p53 binds as a tetramer to its response element (RE) RRRCWWGYYYRRRCWWGYYY (R–A or G, Y–C or T, W–A or T), consisting of two decameric half sites divided further into four pentameric quarter sites (RRRCW or WGYYY). Thus, each p53 monomer binds to each quarter site of the p53 RE. This arrangement allows for some flexibility in the selection of p53 binding sites, which can sometimes deviate from the consensus sequence [4].

Recently, we published the transcriptomic data showing the changes in gene expression in an A549 lung cancer cell line exposed to actinomycin D and nutlin-3a (henceforth abbreviated to A + N) [5]. Earlier, we observed that these two substances synergize in the activation of p53 and some p53-regulated genes [6,7]. The mechanism of synergy is not known. Actinomycin D inhibits RNA polymerase I, which induces nucleolar stress and the release of ribosomal proteins from the nucleolus. Some of these proteins bind to MDM2, which is the negative regulator of p53. MDM2, sequestered by the ribosomal proteins, does not destabilize p53, which accumulates and activates its target genes [8]. More recent studies have added important details to this picture. It could be expected that the depletion of any ribosomal protein triggers ribotoxic stress leading to the formation of unassembled ribosomal components (e.g., uL5, uL18), the sequestering of MDM2, and the accumulation of p53. Unexpectedly, the depletion of any one among two-thirds of the human ribosomal proteins has no significant impact on p53 accumulation. Only one-third of ribosomal proteins are important for p53 homeostasis because their individual depletion increases the p53 level [9]. Thus, the relationship between nucleolar structure and activation of p53 is not as straightforward as one might expect. A more comprehensive picture of the role of p53 in nucleolar stress is given in this review [10].

The nucleolar stress leading to the sequestration of MDM2 is probably only part of the mechanism by which actinomycin D activates p53 because we observed that this drug also promotes p53 phosphorylation by an undefined kinase [11]. Phosphorylation of p53 by various kinases on different amino acids is crucial for the stimulation of p53 as a transcription regulator [12]. MDM2 inhibits p53 by promoting its degradation but also by concealing its transcription-activating domain [13,14]. Nutlin-3a is a molecule that binds to MDM2 and fills its p53 interaction pocket to prevent p53–MDM2 interaction, which in turn results in p53 stabilization and the activation of p53-target genes [15]. We hypothesize that when actinomycin D and nutlin-3a are applied together, they synergize because actinomycin D activates p53-phosphorylating kinases and nutlin-3a prevents MDM2 from covering p53, thereby giving the kinases easy contact with p53 and promoting strong phosphorylation. By employing this p53 activation mode (A + N) together with a cell line (A549) never used before in high-throughput searches for p53-regulated genes, we were able to identify new candidate p53-target genes [5,16].

One of the genes strongly activated by A + N is *DUSP13*, selected by us for detailed study not only because of its activation by A + N (55-fold by RNA-Seq) but also because its expression, as suggested by the location of mapped sequencing reads, starts from an alternative promoter in the intron [5,16]. We hypothesized that p53 activated by A + N induces the expression of *DUSP13* from this alternative promoter. Moreover, *DUSP13* is an example of an intriguing class of genes that are poorly activated by camptothecin, actinomycin D, and nutlin-3a acting solo but are very strongly upregulated when actinomycin D and nutlin-3a are applied together [16].

**Figure 1:**
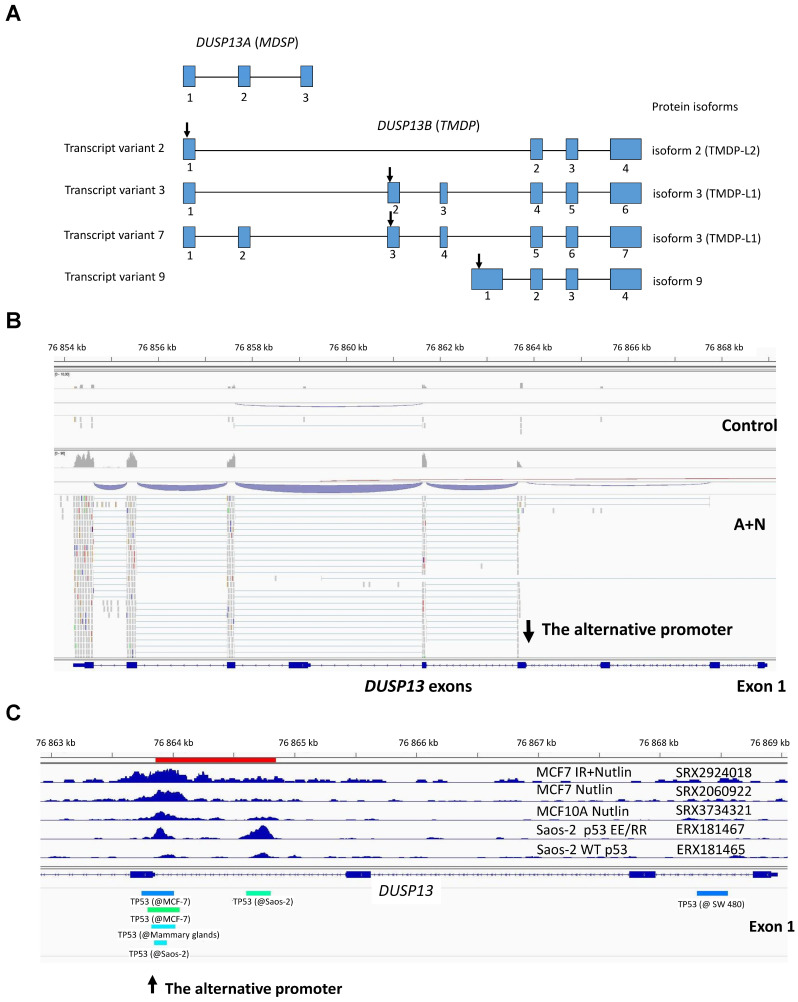
Treatment with A + N activates an alternative promoter in the intron of the *DUSP13* gene. (**A**) The structure of the *DUSP13* locus. The exons are displayed as blue boxes and the starts of open reading frames for individual isoforms are marked as arrows. For clarity, only the relevant transcript variants are shown. The GeneCards database splits *DUSP13* into two genes, *DUSP13A* (alias *MDSP*) and *DUSP13B* (alias *TMDP*). The exons are numbered for each individual transcript variant. Drawn according to data from ncbi.nlm.nih.gov/gene/51207 (accessed on 15 April 2024). (**B**) Genome Browser (IGV) views of RNA-Seq reads mapped to the *DUSP13* gene in mock-treated (control) A549 cells and cells exposed to A + N for 30 h. The raw sequencing data are deposited in the Sequence Read Archive under accession number PRJNA757776. The IGV numbers *DUSP13* exons from right to left. All exons utilized to splice individual transcript variants are shown. (**C**) Genome Browser (IGV) views of p53 binding peaks in the 5′ part of the *DUSP13* gene. Using the ChIP-Atlas tool [19], we imported publicly available coverage tracks from five ChIP-Seq experiments aimed at finding p53 binding sites in the MCF7 cell line exposed to ionizing radiation (IR) and Nutlin (sample ID SRX2924018), MCF7 cells treated with Nutlin (sample ID SRX2060922), MCF 10A cells from non-cancerous breast epithelium exposed to Nutlin (sample ID SRX3734321), the Saos-2 cell line ectopically expressing wild-type p53 (sample ID ERX181465), and Saos-2 cells ectopically expressing pairs of engineered p53 molecules with strong cooperative binding of p53 monomers (sample ID ERX181467). The red, thick horizontal line marks the location of the cloned promoter. The location of ChIP-Seq peaks identified by ChIP-Atlas are shown by horizontal bars at the bottom of the graph.

Initially, *DUSP13* was cloned as a gene coding for a protein abundantly expressed in the testes [17]. The regulation of *DUSP13* is very complicated and unusual, so we considered it interesting to learn how p53 contributes to this regulation. According to early studies, *DUSP13* codes for two isoforms of proteins, which function as dual-specificity phosphatases removing phosphate groups from serines/threonines as well as from tyrosines. *DUSP13* codes these isoforms utilizing the alternative open reading frames (ORF), which is extremely rare in eukaryotes. Exons 1–3 in *DUSP13* code for muscle-restricted dual-specificity phosphatase (MDSP), whereas exons 7–9 encode testis and skeletal-muscle-specific dual-specificity phosphatase (TMDP). However, the GeneCards database considers the DNA fragments coding for *DUSP13* isoforms as separate genes—*DUSP13A* (MDSP) and *DUSP13B* (TMDP). On the other hand, the human genome assembly GRCh38p14 and IGV genome viewer display *DUSP13* as a single gene. This ambiguity is due to the complicated expression of this locus. For instance, the transcript variants 3 and 7, which encode the *DUSP13B* isoform named TMDP-L1, share the exons with *DUSP13A* (Figure 1A). The RT-PCR analysis of RNA from skeletal muscles revealed the existence of mRNAs able to code the isoform TMDP-L1 and another named TMDP-L2; however, the Western blotting analysis of proteins extracted from muscles did not reveal the presence of these proteins [18]. These results generate several questions, e.g., are these mRNA forms translated, was the employed Western blotting method sensitive enough to detect their presence, and, if expressed, what is their physiological function?

Our RNA-Seq data [5,16] revealed that activation of p53 by A + N in lung cancer cells may induce the expression of a DUSP13 isoform related to TMDP. This is an unexpected observation because so far, the expression of *DUSP13* has only been detected in skeletal muscle and the testes. Our results suggest that p53 has the potential to activate the expression of *DUSP13* in other tissues. Interestingly, TMDP was found to deactivate stress-activated kinases named MAPKs [20], which suggests that p53 may modulate the stress response of a cell by activation of this DUSP13 isoform.

## 2. Materials and Methods

### 2.1. Cell Culture and Treatment

A549 (lung adenocarcinoma, American Type Culture Collection—ATCC, Manassas, VA, USA), NCI-H292 (mucoepidermoid pulmonary carcinoma, ATCC), and U-2 OS (osteosarcoma, ATCC) cells were grown as previously described [6]. NCI-H460 (lung cancer, ATCC) was cultured in RPMI-1640 supplemented with 2 mM L-glutamine, 4.5 g/L glucose, 1 mM sodium pyruvate, and 10% heat-inactivated fetal bovine serum (FBS; Invitrogen, Carlsbad, CA, USA). NCI-H1299 large cell carcinoma of the lung cell line (ATCC) was cultured in low-glucose DMEM (with 10% FBS), A375 melanoma cell line (ATCC) was cultured in high-glucose DMEM (with 10% FBS), AGS gastric adenocarcinoma cell line (ATCC) was cultured in McCoy’s 5A medium (with 10% FBS), MCF7 breast adenocarcinoma cell line (ATCC) was cultured in DMEM F12 medium (with 10% FBS), and NCI-H23 lung adenocarcinoma cell line (ATCC) was cultured in RPMI-1640 (with 10% FBS). All culture media were supplemented with 1% penicillin-streptomycin (Sigma-Aldrich, St. Louis, MO, USA). The cells were grown at 37 °C/5% CO_2_.

The stock solutions of chemicals were prepared in DMSO: actinomycin D (10 µM; Sigma-Aldrich, St. Louis, MO, USA), camptothecin (10 mM; Calbiochem-Merck, Darmstadt, Germany), nutlin-3a (10 mM; Selleck Chemicals LLC, Houston, TX, USA), idasanutlin (10 mM; MedChemExpress, Monmouth Junction, NJ, USA), and RG7112 (10 mM; MedChemExpress). Stock solutions were diluted in culture medium to the following concentrations: 5 nM actinomycin D, 5 µM nutlin-3a, 5 µM camptothecin, 5 µM idasanutlin, and 5 µM RG7112. Control cells were mock-treated with a medium containing DMSO.

The generation of p53-deficient A549 and U-2 OS cells using CRISPR/Cas9 technology was described earlier [5].

### 2.2. Semi-Quantitative Real-Time PCR

Total RNA samples were isolated from cells using the RNeasy mini kit (Qiagen, Hilden, Germany). The cDNA was synthesized with MuLV reverse transcriptase and random hexamers (Applied Biosystems, Foster City, CA, USA). Measurements of mRNA levels were performed using Real-Time 2x PCR Master Mix SYBR (A&A Biotechnology, Gdynia, Poland). We used the following primers to measure the expression of *DUSP13* by RT-PCR: 5′-GAT ACA TCC GAG CTG CCC TC-3′ and 5′-GCC TCT ACC AGC GTC ATG TT-3′. The primers for internal control *ACTB* were as follows: 5′-GCA AGC AGG AGT ATG ACG AG-3′ and 5′-CAA ATA AAG CCA TGC CAA TC-3′. Amplifications were performed on a CFX96 Real-Time System (Bio-Rad, Hercules, CA, USA). In each PCR run, cDNA samples were amplified in triplicate. Relative quantitation of mRNA was carried out using the ΔΔCT method with *ACTB* as a reference. Mean and standard deviation were calculated from three or four biological replicates.

### 2.3. Western Blotting

The preparation of the whole-cell lysates using an IP buffer supplemented with protease and phosphatase inhibitors as well as the preparation of the concentrated conditioned medium were described previously [7]. Aliquots of lysates (35–50 µg) were separated by SDS-PAGE on 8% or 13% gels and electro-transferred onto PVDF membranes. Before incubation with the primary antibody, the membranes were incubated for 1 h at room temperature in a blocking solution (5% skim milk in PBS with 0.1% Tween-20). The anti-phospho-Ser37 p53 and anti-phospho-Ser392 p53 antibodies were obtained from Cell Signaling Technology (Danvers, MA, USA). Anti-p53 (DO-1) and the loading control anti-HSC70 (B-6) antibodies were obtained from Santa Cruz Biotechnology (Dallas, TX, USA). Anti-DUSP13 antibody (rabbit polyclonal) was obtained from Proteintech (Rosemont, IL, USA). The HRP-conjugated anti-GFP (green fluorescent protein) antibody (B-2) was obtained from Santa Cruz Biotechnology. All incubations with primary antibodies were performed overnight at 4 °C in a blocking solution. HRP-conjugated secondary antibodies (anti-mouse, anti-rabbit) were detected using chemiluminescence (SuperSignal West Pico or SuperSignal West Femto chemiluminescent substrate, Thermo Fisher Scientific, Waltham, MA, USA).

### 2.4. Molecular Cloning

The alternative promoter region of *DUSP13* was cloned into the pGL3-Basic reporter vector, which encodes firefly luciferase (Promega, Madison, WI, USA). The sequences of primers used to amplify the promoter are 5′-TTTT ACG CGT CCA CCT CTG CTT CCT CTA CA-3′ and 5′-TTTT CTC GAG CCA GCT CTG GAA GAG AGA TGA-3′. The primers were designed to contain the restriction sites for MluI and XhoI (underlined). Amplified DNA was ligated into the respective sites of the pGL3-Basic plasmid. PCR was performed with a PfuPlus! DNA polymerase mix (EURx, Gdańsk, Poland) to ensure high-fidelity DNA amplification. The inserted DNA was sequenced to ensure that the clones contained no mutations.

To generate DUSP13-EGFP chimeric cDNA, we first isolated RNA from A549 cells exposed to A + N and then generated cDNA as described above. We employed a triple-ligation method to clone the cDNA of the DUSP isoform, taking advantage of the ClaI restriction site naturally occurring in the middle of its cDNA sequence. The 5′ part of cDNA was amplified with the following primers: 5′-TTTT AAG CTT ACA GAG CTC ATC TCT CTT CC-3′ and 5′-CAG ACC TC**A TCG AT**A TGG TTC-3′. This PCR product, containing the ClaI site (bold), was digested with HinDIII and ClaI restriction enzymes. The HinDIII was created with PCR primer (underlined). The 3′ part of cDNA was amplified with the following primers: 5′-AAC CAT **ATC GAT** GAG GTC TGG-3′, and 5′-AGG GTC AGG GAT CCT GGC T-3′. This PCR product was digested with ClaI and BamHI (underlined). These products were ligated into the HinDIII and BamHI sites of the pcDNA3.1(+) plasmid. The whole insert was sequenced to ensure that the clone without mutations was selected. In this way, we generated expression vector coding for an unmodified isoform of *DUSP13*. Subsequently, we amplified the cDNA of the *DUSP13* isoform from this plasmid using the following primers: 5′-TTTTGCTAGCCCTGCC**ATG**GGGCTCTGCCAC-3′ and 5′-TTTTAAGCTTTCC**GAA**CCGCCCCGTCTCCCG-3′. In the first primer, the NheI restriction site was created (underlined), and the location of the start codon is marked in bold. In the second primer, the HinDIII site was created (underlined), and the last codon of the *DUSP13* isoform (Phe, in reverse) is marked by bold font. The PCR product was ligated into the NheI and HinDIII sites of the pcDNA3.1(+) plasmid containing EGFP cDNA cloned into the BamHI and EcoRI sites. In this way, we ligated *DUSP13* cDNA in the frame with EGFP cDNA. The last PCR step was needed to remove the stop codon of the *DUSP13* isoform. The correctness of the constructed chimeric gene was confirmed by sequencing. To find out if the fusion protein is expressed, we transfected the plasmid coding for the chimeric protein or for only EGFP to U-2 OS cells using FuGENE 6. The next day, the cells were harvested and the lysates were prepared. The expression of proteins was examined with Western blotting as described above. In order to observe the cellular localization of the fusion protein DUSP13-EGFP (or control EGFP), the U-2 OS cells were seeded on chambered cover glasses, and on the next day, the cells were transfected with the plasmids using FuGENE 6. The localization of proteins in living cells was observed starting 24 h post-transfection with a Zeiss confocal microscope (Carl Zeiss AG, Oberkochen, Germany).

### 2.5. Site-Directed Mutagenesis

The mutations of the CWWG (W–A or T) sequence in the putative p53 response element from the *DUSP13* promoter were created with a GeneArt Site-Directed Mutagenesis PLUS kit (Life Technologies, Carlsbad, CA, USA) using the following oligonucleotides: DUSP13 forward (5′-GGTGACTGGCCTGGG**GCGT**CTTGGGAGCTGGAAC-3′) and DUSP13 complementary reverse (5′-GTTCCAGCTCCCAAG**ACGC**CCCAGGCCAGTCACC-3′) (the sites of mutation are underlined).

### 2.6. Luciferase Reporter Assay

The luciferase reporter assay was performed as described previously [7]. In short, U-2 OS cells were co-transfected using FuGENE 6 (Promega, Madison, WI, USA) with a combination of reporter vector, encoding firefly luciferase under the control of the tested promoter (wild type or mutant), and expression vector pC53-SN3, encoding wild-type p53 or pC53-SCX3 encoding the Val143Ala p53 mutant (a gift from Dr. Bert Vogelstein and Dr. Kenneth W. Kinzler from Johns Hopkins University, Baltimore, MD, USA [21]). As a negative control, the p53 plasmid was replaced by an empty vector. The transfection mixture also contained the pRL-TK vector, encoding *Renilla* sp. luciferase under the control of a herpes simplex virus thymidine kinase (HSV-TK) promoter (internal control). The next day, the cells were washed with culture medium and incubated with fresh medium for an additional 24 h. The cells were lysed with PLB buffer from the Dual-Luciferase Reporter Assay system (Promega, Madison, WI, USA), and the activities of the luciferases were measured. Firefly luciferase activity was normalized against *Renilla* sp. luciferase activity to produce normalized firefly luciferase activity (NFLA). The NFLA in the control cells was set as 1 and the activity in the experimental cells was expressed as its fold-change. Each transfection was performed in triplicate in three independent experiments.

To test how the endogenous p53 impacts on the activity of the cloned *DUSP13* promoter, the cells were transfected with the *DUSP13* reporter vector and the abovementioned pRL-TK control vector. After 24 h, the medium with the transfection mixture (FuGENE 6 + DNA) was removed and the cells were exposed either to the control medium or the medium with A + N to activate the endogenous p53. After 24 h, the activities of both luciferases were measured, and normalized firefly luciferase activity was calculated as described in the previous paragraph.

## 3. Results

The results of high-throughput sequencing of mRNA molecules (RNA-Seq) can easily reveal the alternative splicing and the use of other promoters of genes. In our recently published RNA-Seq results [5,16], we noted that the transcription of the *DUSP13* gene in A549 cells exposed to A + N starts from the alternative promoter located in the intron (Figure 1B). Based on the pattern of exon splicing (Figure 1B), we conclude that the mRNA in the cells exposed to A + N codes for the isoform of DUSP13B called TMDP-L1. The known transcript variants 3 and 7 of *DUSP13B* (Figure 1A), which encode TMDP-L1, contain the long 5′UTR coded by one (variant 3) or two (variant 7) upstream exons shared with *DUSP13A.* In the case of A549 cells exposed to A + N, the transcription starts just upstream of the exon with the translation start codon, so the upstream codons for long 5′UTR are not included in this transcript. We observed a similar expression pattern in RNA-Seq data from three other biological replicates of the experiment (A549 cells exposed to A + N for 30 h) and in three other cell lines (A375, U-2 OS, and NCI-H460) treated in the same fashion [16]. Note that in the transcriptomic data, *DUSP13* upregulation can be found already after 30 h stress. The raw sequencing data are deposited in the Sequence Read Archive under accession numbers PRJNA831359 and PRJNA837373.

The location of p53 binding sites in DNA can be detected using sequencing of DNA isolated from chromatin immunoprecipitated with anti-p53 antibody (ChIP-Seq). The results of these investigations performed on various cells growing in control conditions or exposed to various stress factors have been published in numerous papers. They can be viewed via the ChIP-Atlas platform [19]. Interestingly, we noticed two p53 ChIP-Seq peaks within the intron of *DUSP13*; however, these two peaks were detected only in Saos-2 cells ectopically expressing engineered p53 molecules (p53EE/RR) with strong cooperative binding of p53 monomers (Figure 1C). Wild-type p53 expressed in Saos-2 did not bind to this locus (Figure 1C) [22]. Interestingly, the locus close to the exon was found to be bound by p53 in three data sets: in the MCF7 cell line exposed to nutlin-3a and ionizing radiation, in MCF7 cells exposed to nutlin-3a, and in non-cancerous breast epithelial cells (MCF 10A) exposed to nutlin-3a. Thus, in epithelial cells derived from the breast, p53 activated by nutlin-3a is able to bind to the sequence close to the indicated *DUSP13* exon. The ChIP-Atlas tool does not show p53 binding to this locus in any other examined cell line (except Saos-2 expressing the p53EE/RR mutant). Thus, p53 binds to this site in a cell-type-specific manner, preferentially in cells derived from breast epithelium. To find out if *DUSP13* was found in transcriptomic studies to be activated by nutlin-3a in MCF7 cells, we browsed the database published by Fisher et al. [23]. In five transcriptomic databases, *DUSP13* was found to be activated by nutlin-3a in MCF7 cells. In three of the studies, the upregulation was statistically significant. Thus, in MCF7 cells, nutlin-3a induces both the upregulation of *DUSP13* mRNA and p53 binding to the alternative promoter detected by us.

**Figure 2:**
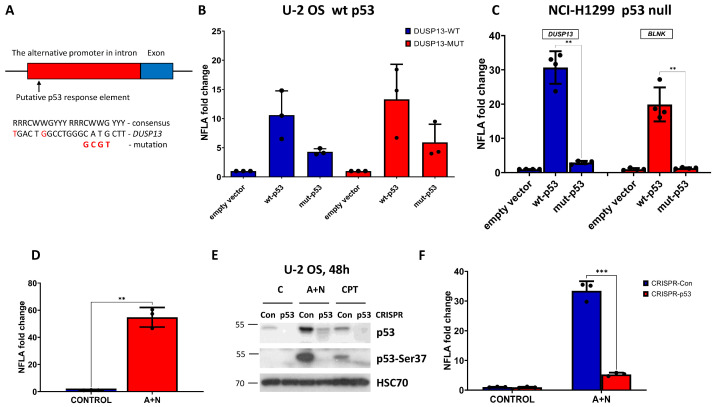
The p53 protein activates the alternative promoter of *DUSP13*. (**A**) The sequence of a putative p53-response element within the alternative *DUSP13* promoter is shown together with the consensus sequence of the response element and the mutations we generated. (**B**) The relative values of NFLA (normalized firefly luciferase activity) in U-2 OS cells transfected with (bars from left to right): plasmid with wild-type *DUSP13* promoter and empty expression vector, plasmid with wild-type *DUSP13* promoter and wild-type p53 expression vector, plasmid with wild-type *DUSP13* promoter and mutant p53 expression vector, plasmid with mutant *DUSP13* promoter and empty expression vector, plasmid with mutant *DUSP13* promoter and wild-type p53 expression vector, and plasmid with mutant *DUSP13* promoter and mutant p53 expression vector. The means and standard deviations from three biological repeats performed in triplicate are shown. (**C**) The relative values of NFLA in p53-null NCI-H1299 cells transfected with reporter plasmids with a cloned *DUSP13* promoter (blue bars) or with the cloned promoter of another p53-regulated gene *BLNK* (red bars). The reporter plasmids were co-transfected with a control empty vector, the vector expressing wild-type p53, or the vector expressing mutant p53. The statistical significance was calculated by multiple unpaired *t*-tests; ** *p* ≤ 0.01. The calculation was performed using GraphPad Prism version 9.5.1 (2023) for Windows, GraphPad Software, Boston, MA, USA, www.graphpad.com, accessed on 15 April 2024. (**D**) The relative value of NFLA in U-2 OS cells transfected with a plasmid with wild-type *DUSP13* promoter growing in control conditions (mock treatment) or exposed to A + N. The results represent means and standard deviations from three biological replicates, and the *p* value was calculated with an unpaired *t*-test, ** *p* ≤ 0.01. The calculation was performed using GraphPad Prism version 9.5.1. (**E**) The expression of total p53, p53 with phosphorylated Ser37 and HSC70 as a loading control in U-2 OS cells with p53 expression knocked-down by CRISPR/Cas 9 (CRISPR-p53), and in control cells for knockdown (CRISPR-Con) growing in control conditions (mock treatment—C) or exposed to A + N or camptothecin (CPT). The expression of indicated proteins was determined by Western blotting. (**F**) The relative values of NFLA in U-2 OS cells transfected with a plasmid with wild-type *DUSP13* promoter. The transfection was performed in p53-deficient cells (CRISPR-p53) and their controls (CRISPR-Con) exposed to A + N for 24 h (A + N) or mock-treated (CONTROL). The results represent means and standard deviations from three biological replicates, and the *p* value was calculated with multiple unpaired *t*-tests; *** *p* ≤ 0.001. The calculation was performed using GraphPad Prism version 9.5.1.

In order to find out if this fragment of intron can function as a promoter activated by p53, we cloned it into the reporter vector. The cloned fragment encompasses the region of the two p53 ChIP-Seq peaks as marked in Figure 1C (red line). Moreover, we noticed that there is a sequence closely matching the consensus site of the p53 response element and overlapping the distal peak (Figure 2A). The *DUSP13* sequence shows only two mismatches, making this sequence a highly probable p53 response element (Figure 2A). We mutated its four critical sequence positions, as shown in Figure 2A. The reporter assays performed on the U-2 OS cell line demonstrated that wild-type p53 stimulated the wild-type promoter approximately 10-fold (Figure 2B). The activation of the promoter by the mutant p53 was significantly weaker. However, the mutant promoter can still be activated by wild-type p53, and hence, the p53-response element must be located elsewhere in the cloned region, probably within the proximal ChIP-Seq peak. Because U-2 OS cells have endogenous wild-type p53, which could interfere with the ectopically expressed p53 molecules, we performed the reporter assay on a cell line, NCI-H1299, which does not express endogenous p53. Moreover, for comparison, we employed a reporter plasmid with another p53-activated promoter cloned by us earlier from the *BLNK* gene [5]. This gene was also identified by others as a p53-regulated gene [24]. The reporter test (Figure 2C) performed on the p53-null cell line confirmed our conclusion that ectopically expressed wild-type p53 strongly activated the cloned *DUSP13* promoter while the mutant p53 lost this ability. Moreover, we found that activation of the *DUSP13* promoter was stronger than the *BLNK* promoter.

Next, we tested if the cloned *DUSP13* promoter can be activated by the endogenous p53. To this end, U-2 OS cells were transfected with the reporter vector containing the wild-type *DUSP13* promoter controlling the firefly luciferase gene (Figure 2D). As an internal control, we transfected a reporter vector with the gene for the luciferase from *Renilla* sp. transcriptionally regulated by the HSV-TK promoter to control for transfection efficiency and stress induced by A + N. Twenty-four hours after transfection, the control cells were mock-treated, whereas the experimental cells were exposed to A + N for 24 h. Subsequently, the cells were harvested, and the activities of both luciferases were measured. The firefly luciferase activity was divided by *Renilla* luciferase activity producing the normalized firefly luciferase activity (NFLA). In cells exposed to A + N, the value of NFLA increased more than 50-fold, indicating that exposure to A + N stimulates the activity of the cloned promoter, probably by activating the endogenous p53 (Figure 2D). To test the involvement of p53, we performed this experiment on p53-deficient U-2 OS cells and their controls prepared using CRISPR/Cas9 technology as described by us earlier [5]. To show that these cells are p53-deficient (Figure 2E), we exposed them to A + N or camptothecin (a precursor of the anticancer drugs—topotecan and irinotecan) and examined the expression of total p53 or its form with phosphorylated Ser37—the amino acid located in the transcription-activating domain, a fragment, which is a target of CRISPR/Cas9-generated DNA breaks. The Ser37 is phosphorylated on activated p53. The p53-deficient cells express a low amount of total p53 and p53 with the activating phosphorylation on Ser37 (Figure 2E). In control cells for knockdown, the exposure to A + N activates the *DUSP13* promoter more than 30-fold, whereas in p53-deficient cells, it is only five-fold (Figure 2F). Thus, the activity of p53 is required to strongly activate the alternative promoter of *DUSP13* following exposure to A + N.

**Figure 3:**
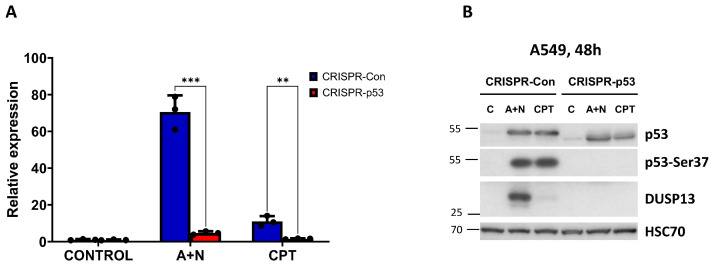
Expression of DUSP13 measured at mRNA and protein levels is attenuated in p53-deficient cells. (**A**) The expression of *DUSP13* mRNA was measured with semi-quantitative RT-PCR in mock-treated cells (CONTROL) or cells exposed to A + N or camptothecin (CPT) for 30 h. The p53 knockdown was performed with CRISPR/Cas9 technology. The results represent mean and standard deviations from three biological replicates, and the *p* values were calculated with multiple *t*-tests; ** *p* ≤ 0.01, *** *p* ≤ 0.001. The calculations were performed using GraphPad Prism version 9.5.1 (2023) for Windows. (**B**) The expression of p53, its form with phosphorylated Ser37 and DUSP13 in p53-deficient cells (CRISPR-p53), and their controls (CRISPR-Con) were prepared as described in (**A**) and exposed to A + N or camptothecin (CPT) for 48 h or mock-treated (C). The expression of indicated proteins was determined with Western blotting.

To find out if the activation of the endogenous *DUSP13* gene is controlled by p53, we performed an experiment with p53-deficient A549 cells prepared by CRISPR/Cas9 technology as described previously [5]. This is a mixture of p53-null clones and clones with a deletion in the transcription-activating domain of p53. The p53-deficient cells and their controls were exposed to A + N or another strong activator of p53-camptothecin. After exposing cells to these compounds, either RNA samples were isolated for RT-PCR or protein lysates were prepared for Western blotting. Both analyses gave concordant results, namely that A + N induces stronger activation of *DUSP13* than camptothecin, and that wild-type p53 is indispensable for the activation of *DUSP13* by either compound (Figure 3A,B). This Western blot also shows that the protein coded by the alternative mRNA of *DUSP13* is actually produced in cells exposed to A + N (or camptothecin) and can be detected by the antibody employed in our experiments (Figure 3B).

**Figure 4:**
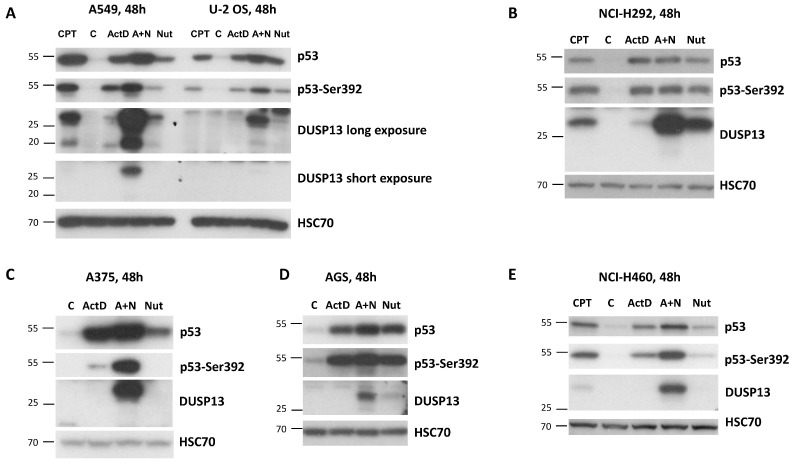
The expression of DUSP13 is induced by A + N in all examined cell lines with the wild-type p53 gene. (**A**–**E**) Western blots showing expression of indicated proteins in selected cell lines with wild-type p53 mock-treated (C) or incubated for 48 h with actinomycin D (ActD), nutlin-3a (Nut), both substances (A + N), or camptothecin (CPT).

Subsequently, using Western blotting, we examined the expression of DUSP13 in various cancer cell lines exposed to actinomycin D, nutlin-3a, A + N, and camptothecin. We selected three lung cancer cell lines (A549, NCI-H460, and NCI-H292), the osteosarcoma cell line (U-2 OS), the melanoma cell line (A375), and the gastric adenocarcinoma cell line (AGS). All cell lines had wild-type gene coding for the p53 protein. The cells were exposed to the compounds for 48 h (Figure 4). Based on the results of our earlier experiment, we selected this time span as optimal to detect strong protein accumulation in cells exposed to A + N [7]. As a surrogate marker of p53 activation, we used the phosphorylation status of Ser392. In our previous study of A549 cells, the amount of phospho-Ser392-p53 correlated well with the expression level of the examined p53 target genes [7]. What is clearly visible on the Western blots is a very strong synergy between actinomycin D and nutlin-3a in the activation of the *DUSP13* gene in all cell lines tested, and weaker activation of the gene by camptothecin. This is consistent with our transcriptomic results [16]. The DUSP13 protein is barely detectable in cells exposed to actinomycin D or nutlin-3a acting alone, whereas the accumulation of DUSP13 in cells exposed to both compounds is very strong. The exception is the NCI-H292 cell line showing relatively high expression of DUSP13 protein, even in cells exposed to nutlin-3a acting solo. This is consistent with the aforementioned observations made on MCF7 cells—that in some cell types, this gene can be upregulated by “standard” activation of p53 with nutlin-3a. In some cells, we observed a striking correlation between the phosphorylation of p53 on Ser392 and the expression of DUSP13 (A375), whereas in other cells, the correlation was either weak or absent. So, the phosphorylation status of Ser392 is not a perfect indicator of the capacity of p53 to activate the *DUSP13* gene. In order to explain the divergent ability of p53 to activate the *DUSP13* gene, one would need to perform a detailed analysis of the abundant posttranslational modifications of p53, which modulate its activity [25]. The general understanding of the mechanisms that determine the cell specificity of gene activation by p53 is very limited. *DUSP13* may be one of the models for such studies.

Regular exposure to our blots detects the DUSP13 form of a size of approximately 30 kDa (Figure 4). After long exposure to the signals on the blot, another protein is detected by the employed antibody in A549 cells treated with A + N. This protein form has a molecular mass of approximately 20 kDa compared with the approximately 30 kDa of the major form (Figure 4A). We suspect that it may be DUSP13 translated from an AUG codon downstream of the major translation start site (see below). There is a possibility that the shorter protein is the TMDP-L2 isoform of DUSP13B, which has a calculated size 5 kDa smaller when compared with TMDP-L1. However, this possibility appears very unlikely for the following reason: the translation of TMDP-L2 starts from the codon located in the first exon (Figure 1A), whereas our RNA-Seq data show that this exon is not transcribed in cells exposed to A + N (no sequence reads mapped to this exon, Figure 1B). A similar pattern was observed in the aforementioned RNA-Seq data from additional biological replicates of the treated A549 cells and from other treated cell lines (U-2 OS, A375, and NCI-H460) [16].

**Figure 5:**
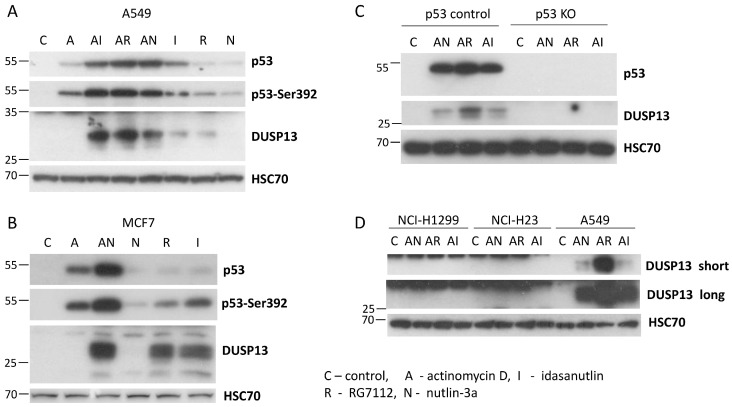
The other antagonists of MDM2–p53 interaction, idasanutlin, and RG7112 also synergize with actinomycin D in inducing the expression of DUSP13. (**A**) A549 cells were exposed for 48 h, as shown, to various combinations of actinomycin D (A) and the antagonists of MDM2–p53 interaction: nutlin-3a (N), idasanutlin (I), and RG7112 (R). The expression of the indicated proteins was determined with Western blotting. (**B**) MCF7 cells were exposed for 48 h to actinomycin D, actinomycin D with nutlin-3a, and to the antagonists of MDM2–p53 interaction acting alone. (**C**) The expression of p53 and DUSP13 proteins in the control clone of p53-proficient A549 cells and in the clone of p53-knockout cells (p53 KO), mock-treated (C), or exposed to indicated drug combinations. (**D**) The expression of DUSP13 in p53 wild-type cells (A549), in p53 mutant cells (NCI-H23), and in p53-null cells (NCI-H1299). The cells were mock-treated (C) or exposed to combinations of actinomycin D and the antagonists of MDM2-p53 interaction. To visualize DUSP13, we performed two exposures (long and short) to photosensitive film.

To find out if other antagonists of p53-MDM2 interaction have a similar impact on DUSP13 expression as nutlin-3a, we exposed A549 cells to idasanutlin or RG7112 (reviewed by Kocik et al. [26]) either alone or in combination with actinomycin D. As is demonstrated on Figure 5A, these two compounds also synergize with actinomycin D in the activation of DUSP13. Moreover, at the concentration used in the experiment (5 µM), they induce DUSP13 also when acting alone (Figure 5A).

Because the published high-throughput experiments demonstrated that nutlin-3a can activate *DUSP13* in MCF7 cells [23], we exposed them to actinomycin D alone or in combination with nutlin-3a as well as to the MDM2 antagonists acting unassisted (RG7112, and idasanutlin) (Figure 5B). As expected, the A + N combination upregulated DUSP13; however, nutlin-3a had no detectable effect, whereas both RG7112 and idasanutlin were able to stimulate the expression of the DUSP13 protein. This observation indicates that the isoform of DUSP13, which we detect by the employed antibody, can be induced not only by a drug combination like A + N but also by at least two MDM2 antagonists acting solo. Moreover, as in the previous experiment, it is clear that the more advanced versions of MDM2 antagonists, idasanutlin and RG7112, are better activators of the p53 target DUSP13 than nutlin-3a.

Next, we used these advanced antagonists of the p53–MDM2 interaction to test if the DUSP13 protein can be upregulated in cells in which p53 was deactivated by various mechanisms. First, we tested a p53 knockout clone isolated from the mixture of p53-deficient clones used for the experiment presented in Figure 3. The control clone expresses p53 (Figure 5C). In the p53 knockout clone, neither drug combination upregulated the expression of the DUSP13 protein. Neither was DUSP13 upregulated in lung cancer cell lines, which are either p53 null (NCI-H1299) or express mutant p53 (NCI-H23) (Figure 5D) [27]. Thus, DUSP13 is not upregulated in engineered p53-deficient cells (Figure 3), in p53-null-cells (Figure 5C), or in naturally occurring p53-null or p53-mutated cancer cell lines (Figure 5D).

**Figure 6:**
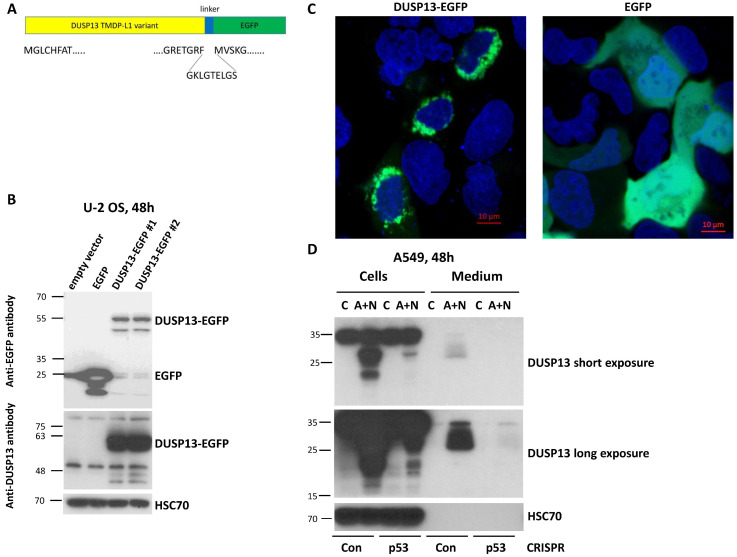
The DUSP13 isoform with the attached enhanced green fluorescent protein localizes to the perinuclear region of U-2 OS cells. (**A**) The structure of the fusion protein expressed from the engineered plasmid. The variant of DUSP13 is fused by the plasmid-coded linker to the enhanced green fluorescent protein (EGFP). The amino acid sequences of the DUSP13 N- and C-ends, the linker, and the start of EGFP are shown. (**B**) The Western blot showing the expression of EGFP and DUSP13-EGFP fusion protein expressed from two clones of the plasmid. The positions of the molecular weight markers (kDa) are placed on the left. The expected size of the unmodified DUSP13 variant is 32 kDa. Two separate blots were probed either with an anti-EGFP antibody or with an anti-DUSP13 antibody. (**C**) The localization of the proteins expressed from the plasmids transfected to U-2 OS cells. The living cells were observed using a Zeiss confocal microscope 24 h after the start of transfection. The nuclei were stained using Hoechst 33342. (**D**) The expression of DUSP13 in cells and in the concentrated medium of cells growing in the control condition (mock treatment, C) or exposed to A + N for 48 h. We used p53 knockdown cells (CRISPR-p53) and the controls for knockdown with wild-type p53 (CRISPR-Con). The expression of indicated proteins was determined with Western blotting. Both panels show the same blot with short (upper) and long (bottom) exposure times.

To gain further insight into the biological function of the TMDP-L1 isoform of DUSP13, we amplified its cDNA sequence from A549 cells exposed to A + N and cloned it into an expression vector in front of the coding sequence of the green fluorescent protein EGFP (Figure 6A). This chimeric cDNA codes for the chimeric protein with EGFP attached to the C-terminal fragment of TMDP-L1. We wanted to keep the native form of the N-terminus of DUSP13 because it may contain a signaling sequence guiding the protein to its destination within a cell or extracellular space. Subsequently, we transfected the vector with the chimeric gene to U-2 OS cells and observed the molecular weight of the fusion protein using Western blotting (Figure 6B) and its localization by confocal microscopy of living cells (no fixation) (Figure 6C). According to the Western blotting (Figure 6B), the size of the DUSP13–EGFP fusion protein (55 kDa) is about 30 kDa higher when compared to the size of EGFP alone (25 kDa), which is in agreement with the expected size of this version of DUSP13 (32 kDa). The fusion protein was detected by an antibody directed against EGFP as well as by an antibody directed against the DUSP13 used in this work (Figure 6B). The microscopic observations demonstrated that the DUSP13–EGFP fusion protein is localized in the cytoplasmic region around the nucleus, in contrast with the localization pattern of EGFP, which localizes in the cytoplasm and nucleus without any aggregation in the perinuclear region (Figure 6C).

The N-terminus of TMDP-L1 contains a sequence that was predicted with high probability as a signal peptide MGLCHFATLALILLVLLEALAQAD [28]. This peptide targets a protein to the secretory pathway encompassing the endoplasmic reticulum, Golgi apparatus, trans-Golgi network, secretory vesicles, and plasma membrane. To find out if this protein is secreted, we analyzed both the cell lysates and conditioned media from control cells and cells exposed to A + N for 48 h. We performed the experiment on the aforementioned p53-deficient A549 cells and their controls. The proteins from the cell lysates and concentrated media were separated by electrophoresis, transferred to membranes, and probed with an anti-DUSP13 antibody. In the lysates, the antibody detected two protein forms (approximately 20 kDa and 30 kDa) but only in p53-proficient cells exposed to A + N (Figure 6D). In the medium, a smear of protein with a size of about 30 kDa and higher was detected, but again, only in the case of p53-proficient A + N-treated cells. In the medium, the 20 kDa protein was not visible even after the overexposure of the detection film (Figure 6D). If this shorter protein is translated from an alternative downstream start codon, then it lacks the signal peptide for secretion. Moreover, the lack of 20 kDa protein in the medium suggests that DUSP13 does not passively spill to extracellular space—in this case, both forms would be detected. In conclusion, a fraction of the endogenous DUSP13 protein from the A549 cells exposed to A + N is secreted to the extracellular space.

## 4. Discussion

The expression of DUSP13 dual-specificity phosphatase is best studied in mice. Exons 1–3 code for the open reading frame of the MDSP isoform expressed in the diaphragm and skeletal muscles, whereas exons 7–9 code for the open reading frame of the TMDP isoform expressed in the testes. The transcript for MDSP also contains exons 4, 5, 7, 8, and 9, which encode long 3′-UTR [17]. Genes encoding alternative ORFs producing two distinct proteins are rare in higher eukaryotes. The isoform expressed in A549 cells exposed to A + N is similar to TMDP. This transcript was found for the first time in muscles and its protein product was named TMDP-L1; however, it was not detected with Western blotting [17]. We found that the expression of TMDP-L1 is induced in A549 cells exposed to A + N. In these conditions, A549 cells produce a protein with the size expected for TMDP-L1 (approximately 30 kDa), which can be detected by the antibody employed in our experiments. The DUSP13 mRNA and protein can also be upregulated by another substance strongly activating p53-camptothecin, although in this case, the upregulation is significantly weaker. Moreover, in some cancer cell lines, the isoform can be strongly upregulated by nutlin-3a or by more advanced forms of MDM2 antagonists acting alone. Activation of *DUSP13* measured at the mRNA or protein levels is strongly attenuated in p53-deficient cells (or prevented in p53-null cells), which indicates that p53 plays a role in this process, apparently in a direct manner because both ectopically expressed and endogenous p53 is able to activate the cloned alternative promoter of *DUSP13*. However, there must be something special in the interaction between p53 and the alternative promoter of *DUSP13* driving the expression of TMDP-L1. The data published by Schlereth et al. [22] demonstrated that in Saos-2 cells, this DNA fragment shows two p53 binding sites: one closer to the first exon (proximal site) and one more distant from the exon (distal site). However, these sites are occupied in Saos-2 cells not by wild-type p53 but only by the artificially prepared mutant version of this protein (Figure 1C), which promotes cooperative binding between p53 monomers. Interestingly, ChIP-Seq analyses performed by others demonstrated that the proximal site is occupied by endogenous p53 in the MCF7 breast cancer cell line exposed to nutlin-3a or in the non-cancerous breast epithelial cell line (MCF 10A) exposed to nutlin-3a. We performed a mutation of a plausible p53 response element overlapping the distal site identified in silico by Tebaldi et al. [4]; however, the mutation did not destroy the ability of the cloned promoter to respond to p53. Thus, this cloned promoter contains other, less obvious p53 binding sites, probably overlapping the proximal ChIP-Seq peak occupied by p53 in cells derived from breast epithelium exposed to nutlin-3a. In all the examined cell lines, actinomycin D and nutlin-3a strongly synergized in the stimulation of the expression of the DUSP13 protein. Actinomycin D or nutlin-3a acting alone did not stimulate the expression of *DUSP13*, the exception being NCI-H292 cells, where this isoform is induced by nutlin-3a (Figure 4B). Thus, “regular” activation of p53 is able to stimulate the expression of *DUSP13* only in subsets of cells (MCF7, MCF 10A, NCI-H292). Therefore, to activate the expression of this isoform of DUSP13, p53 requires a special set of modifications or interactions with other proteins expressed only in some cells. It is also plausible that this alternative promoter is in closed chromatin in most cells and only the treatment with A + N is able to open this chromatin for p53 binding. Interestingly, a recent review by Fischer et al. [23] provides a summary of transcriptomic studies searching for p53-regulated genes. In these analyses, *DUSP13* was identified as a p53 target in only 12 out of 57 studies. However, it must be stressed here that there are p53-regulated genes belonging to the p53 core transcriptional program (approximately 100 members), which are activated in most cell types, while other, more numerous genes are activated by p53 in a cell-specific manner [29]. Apparently, *DUSP13* belongs to the second group, which does not depreciate its role in cellular physiology.

There is another intriguing point that has to be mentioned. All substances that we have employed in our experiments are somehow involved in the induction or reaction to the nucleolar stress. For instance, actinomycin D preferentially intercalates into DNA fragment coding for ribosomal RNA, while camptothecin inhibits topoisomerase I, which is crucial for transcription. Consequently, both compounds lead to inhibition of ribosomal RNA production by RNA polymerase I. On the other hand, nutlin-3a, RG7112, and idasanutlin disrupt p53–MDM2 interaction that mimics the activation of p53 during nucleolar stress, which involves sequestration of MDM2 by selected components of ribosomes leaking from the disrupted nucleoli (reviewed in Ref. [30]). The intriguing question is by what mechanism the combination of two substances involved in the nucleolar stress, actinomycin D and nutlin-3a, induces such spectacular upregulation of *DUSP13* and other p53 target genes [16].

DUSP13 is not a well-studied protein. At the time of writing, entering the phrase “DUSP13” in PubMed only returns 21 papers (18 June 2024). The crystal structure of the TMDP isoform was determined [31]. Interestingly, the TMDP variant, when overexpressed with DUSP4 in the A549 cell line, attenuates TGFβ1-induced migration and drug resistance [32]. In cardiomyocytes, both DUSP13A and DUSP13B were found to reduce cell death induced by H_2_O_2_, although the authors employed *DUSP13B* transcript variant 8 [33], coding for isoform 6, also known as TMDP, which does not contain the N-terminus of the isoform, which was studied in our work. Recently, *DUSP13* was found to belong to a three-gene signature that can accurately distinguish COVID-19 patients from healthy controls [34]. This finding indicates that *DUSP13* may participate in some immune-related activity. Other recently published data indicate that *DUSP13* is a protein that can be detected in blood plasma and may be associated with an increased risk of atrial fibrillation [35]. The expression of *DUSP13* was reduced after epithelial-mesenchymal transition of the ovarian cancer cell line triggered by TGFβ1 [36]. The oncology research focusing on *DUSP13* is very limited [32,36,37,38,39]. Some researchers stumbled upon *DUSP13* during various high-throughput studies. In the latest of these reports, the authors found that a regulatory axis involving lncRNA PVT1, miR-378c, and *DUSP13* is involved in microvascular invasion in hepatocellular carcinoma. Moreover, based on various types of data analyses, the authors suggested that *DUSP13* may be involved in lipid metabolism, the glycosyl compound metabolic process, and the xenobiotic metabolic process [39]. In another study, DUSP13 protein in plasma was found to be significantly associated with gastroesophageal reflux disease [40]. Our analysis suggests that *DUSP13* easily responds to the activation of p53 in cells derived from breast epithelium.

The physiological role of the TMDP-L1 isoform upregulated by p53 is not known. The TMDP expressed in the testes inhibits stress-activated MAPK kinases and suppresses AP-1-dependent gene expression [20]. Whether a similar role is played by the TMDP-L1 variant induced by A + N in a p53-dependent manner remains to be determined. Our data clearly indicate that this isoform of DUSP13 is a part of the p53-regulated stress-response system, even if p53 activates it indirectly. One of the major findings of this paper is that DUSP13 expression is not limited to the testes or muscles but can be found in cancer cells with wild-type p53 exposed to clinically relevant substances (e.g., camptothecin), experimental drug combination (A + N), or antagonists of MDM2–p53 interaction (nutlin-3a, idasanutlin, and RG7112). This line of research may be continued because there is an indispensable tool—the antibody, which can detect TMDP-L1 expression. It is important because, in our experience, there is a scarcity of commercially available and usable antibodies recognizing poorly studied proteins.

## 5. Conclusions

The treatment of A549 lung cancer cells with actinomycin D and nutlin-3a (A + N) induces the expression of the TMDP-L1 variant of *DUSP13* dual-specificity phosphatase governed by the alternative promoter located in the intron. The cloned alternative promoter can be activated by both ectopically expressed and endogenous p53 proteins. In p53-deficient cells, the activation of *DUSP13* is attenuated. The protein of the expected size can be detected with Western blotting in various cancer cell lines with wild-type p53 exposed to A + N, nutlin-3a, or more advanced antagonists of MDM2—RG7112 and idasanutlin. *DUSP13* protein can also be found in the culture medium of exposed cells. The DUSP13 TMDP-L1 variant with the attached green fluorescent protein localizes in the perinuclear region of cells. Thus, the expression of *DUSP13* is not limited, as observed earlier, to skeletal muscles or the testes but can also be induced by activated p53 in cancer cells, thereby indicating that it can be an important element of the p53-dependent stress-response system.

## Data Availability

The sequencing files (fastq) used to generate Figure 1B are available at the Sequence Read Archive under the accession number PRJNA757776. Other processed sequencing data are available from the corresponding author upon request.
